# Data on the catalytic CO oxidation and CO_2_ reduction durability on gC_3_N_4_ nanotubes Co-doped atomically with Pd and Cu

**DOI:** 10.1016/j.dib.2019.104495

**Published:** 2019-09-10

**Authors:** Kamel Eid, Aboubakr M. Abdullah

**Affiliations:** Center for Advanced Materials, Qatar University, P.O. Box 2713, Doha, Qatar

**Keywords:** CO_2_ reduction, CO oxidation, Durability, Gas conversion reactions, One-dimensional gC_3_N_4_, Nanotubes

## Abstract

Understanding the fabrication mechanism of graphitic carbon nitride (gC_3_N_4_) nanostructures is critical for tailoring their physicochemical properties for various catalytic applications. In this article, we provide deep insights into the optimized parameters for the rational synthesis of one-dimensional gC_3_N_4_ atomically doped with Pd and Cu denoted as (Pd/Cu/gC_3_N_4_NTs) and its fabrication mechanism. This is in addition to the CO oxidation durability along with the electrochemical and photoelectrochemical CO_2_ reduction durability of Pd/Cu/gC_3_N_4_NTs. The presented herein results are correlated to the research article entitled “Precise Fabrication of Porous One-dimensional gC_3_N_4_ Nanotubes Doped with Pd and Cu Atoms for Efficient CO Oxidation and CO_2_ Reduction (Kamel Eid et al., 2019).

**Table:** Specifications Table

Subject area	Chemistry
More specific subject area	Catalysis
Type of data	Scheme, Images, Table, and Figures
How data was acquired	Transmission electron microscope ((TEM), TecnaiG220, FEI, Hillsboro, OR, USA), scanning electron microscope ((SEM), Hitachi S-4800, Hitachi, Tokyo, Japan), X-ray diffraction patterns ((XRD), X'Pert-Pro MPD, PANalytical Co., Almelo, Netherlands), CO oxidation (online gas analyzer IR-200, Yokogawa, Japan), CO_2_ reduction Gamry electrochemical analyzer (reference 3000, Gamry Co., USA).
Data format	The obtained data are imaged and analyzed.
Experimental factors	The thermal CO oxidation stability tests were measured under continuous gas mixture flow while heating (25–300 °C). The electrocatalytic CO_2_ reduction durability tests were benchmarked at the room temperature in 0.5 M NaHCO_3_ solution.
Experimental features	Changing the reaction parameters and conditions to optimizing the fabrication process of Pd/Cu/gC_3_N_4_. Investigation the thermal CO oxidation durability as well as the electrochemical and photoelectrochemical CO_2_ reduction of Pd/Cu/gC_3_N_4_. These results are beside the structural and compositional analysis of Pd/Cu/gC_3_N_4_ after the catalytic durability reactions.
Data source location	Center for advanced materials, Qatar University, Doha 2713, Qatar.
Data accessibility	The data are obtained and provided in this article.
Related research article	Eid et al., Precise Fabrication of Porous One-dimensional gC_3_N_4_ Nanotubes Doped with Pd and Cu Atoms for Efficient CO Oxidation and CO_2_ Reduction, *Inorganic Chemistry Communications*.” [1]

**Table:** 

**Value of the data** • Optimization of the fabrication process of gC_3_N_4_ nanostructures doped with binary metals is essential in various catalytic applications. • Understanding the fabrication mechanism of Pd/Cu/gC_3_N_4_NTs is essential for tailoring their physicochemical and catalytic properties for various applications. • The catalytic CO oxidation and CO_2_ reduction durability of Pd/Cu/gC_3_N_4_NTs are central factors in commercial applications. • These data may open new avenues on using gC_3_N_4_-based materials for gas conversion reactions.

## 1 Data

The presented data article is associated with the research article (Kamel Eid et al., 2019 [1]). This includes (i) the SEM and TEM images of metal-free gC_3_N_4_, (ii) the TEM images of Pd/Cu/gC_3_N_4_ prepared in different morphologies, (iii) the CO oxidation durability of Pd/Cu/gC_3_N_4_NTs, Pd/gC_3_N_4_NTs, and Cu/gC_3_N_4_NTs, (iv) the electrocatalytic and photoelectrochemical CO_2_ reduction of Pd/Cu/gC_3_N_4_NTs, and (v) the XRD, EDX, and TEM image of Pd/Cu/gC_3_N_4_ after the CO durability testes.

## 2 Experimental design, materials, and methods

### 2.1 CO oxidation

We tested the CO oxidation reaction in a fixed bed quartz tubular reactor connected to an online gas analyzer (IR200, Yokogawa, Japan) in the presence of 50 mg of each catalyst. Initial pretreatment was carried out at 250 °C under an O_2_ flow of 50 mL min^−1^ for 1 h, and then H_2_ (30 mL min^−1^) for 1 h. Following that, the catalysts were exposed to the gas mixture involving of 4% CO, 20% O_2_, and 76% Ar with a total flow of 50 mL min^−1^ under continuous heating from 25 °C to 400 °C (5°min^−1^) [1], [2], [3], [4], [5]. The percentage of CO conversion (% CO) was calculated using the following equation:

(1) $$\text{\%CO}=\left[\left({\text{CO}}_{\text{in}}-\;{\text{CO}}_{\text{out}}\right)\right]/{\text{CO}}_{\text{in}}\text{×}100$$

where CO_in_ is, the input quantity and CO_Out_ is the output quantity.

### 2.2 Electrochemical reduction of CO_2_

The cyclic voltammogram (CVs), linear sweep voltammogram (LSV), and electrochemical impedance spectroscopy (EIS) measurements were measured on Gamry electrochemical analyzer (reference 3000, Gamry Co., USA) using a three-electrode system composed of a Pt wire (counter electrode), Ag/AgCl (reference electrode), and glassy carbon ((5mm) working electrode). The CVs, LSVs, and EIS were measured in a CO_2_-saturated aqueous solution of 0.5 M NaHCO_3_ at a sweep rate of 50 mV s^−1^. In the photoelectrochemical measurements, the light source was ozone-free xenon lamp (100 W, Abet Technologies, USA) with fluorine-doped tin oxide as a working electrode in a Quartz photo-glass cell (50 mm × 50 mm). The catalyst loading amount of each catalyst on the working electrode was fixed to 10 μg cm^−2^ using. After deposition of each catalyst on the working electrodes, a 5 μL of Nafion solution (1 wt %) was added on each electrode and left to dry completely under vacuum at 80 °C before the measurements.

**Scheme 1:**
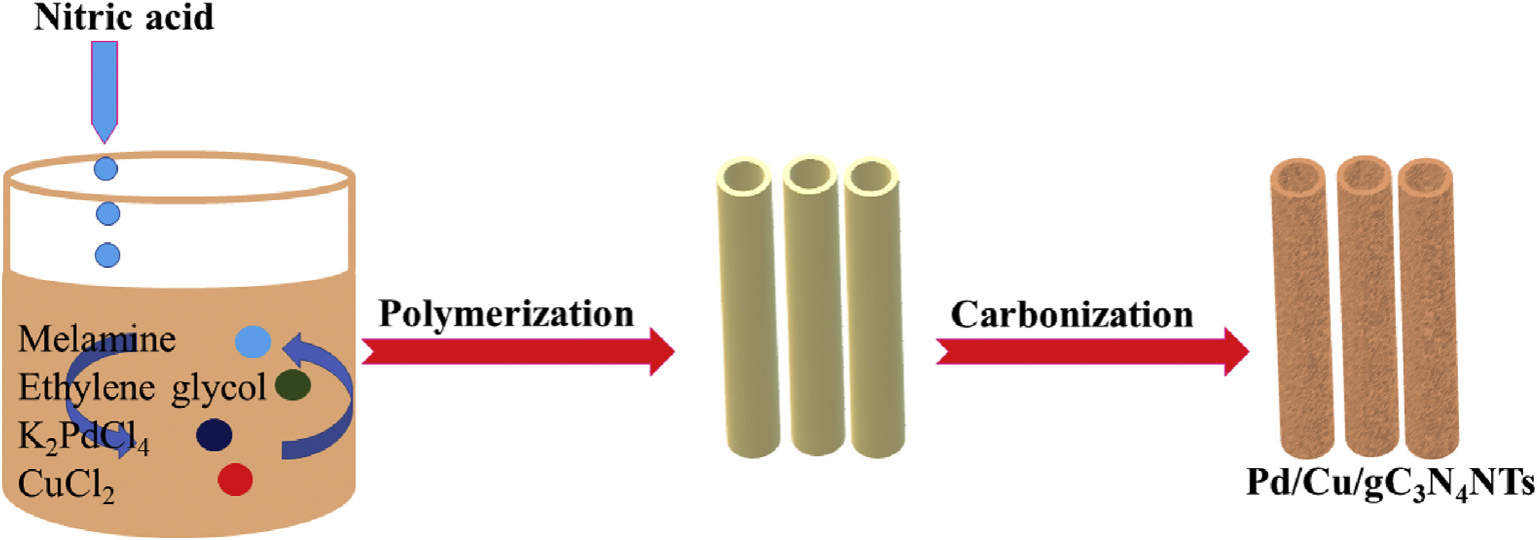
Schematic shows the synthesis process of Pd/Cu/gC_3_N_4_NTs.

Scheme 1 shows the fabrication process of Pd/Cu/gC_3_N_4_NTs, including the initial slow mixing of melamine in an aqueous solution of ethylene glycol solution, contains Pd- and Cu precursors [3]. Then, nitric acid was added dropwise to slowly deprotonate melamine and facilities the polymerization step to polymeric gC_3_N_4_, followed by annealing at elevated temperature to allow the carbonization process and formation of gC_3_N_4_NTs doped with Pd and Cu.

**Fig. 1:**
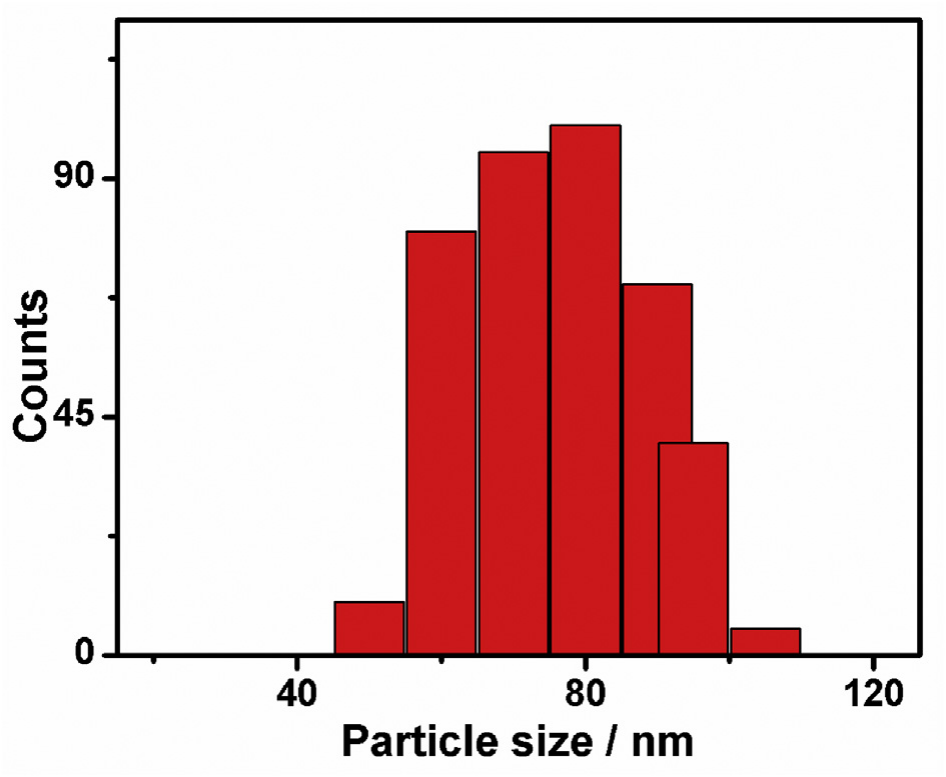
The size distribution histogram of Pd/Cu/gC_3_N_4_NTs.

Fig. 1 shows the histogram chart of Pd/Cu/gC_3_N_4_NTs. The widths of thus obtained Pd/Cu/gC_3_N_4_NTs ranged from 60 to 90 nm. The average width of thus formed nanotubes is nearly 80 nm.

**Fig. 2:**
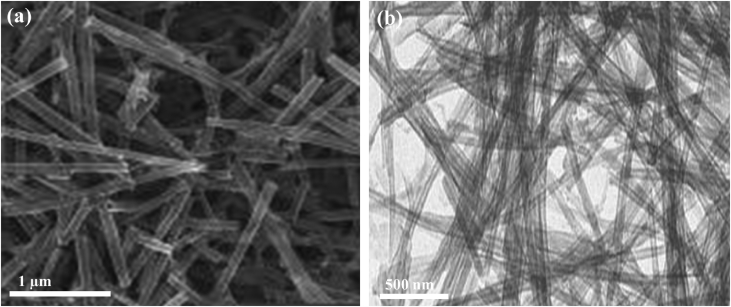
(a) SEM and (b) TEM images of gC_3_N_4_NTs.

Fig. 2 shows the SEM and TEM images of metal-free gC_3_N_4_NTs that were prepared by the same method of Pd/Cu/gC_3_N_4_NTs but in the absence of Pd and Cu precursors. Fig. 2a reveals the SEM image of gC_3_N_4_NTs formed in high yield (nearly 100%) of nanotubes shape. The nanotube shape was uniform and mono distributed with an average width of 78 nm and an average length of 1.4 μm. The TEM image shows the absence of any undesired nanostructures such as spherical nanoparticles or other shapes.

### 2.3 Fabrication parameters optimization

**Fig. 3:**
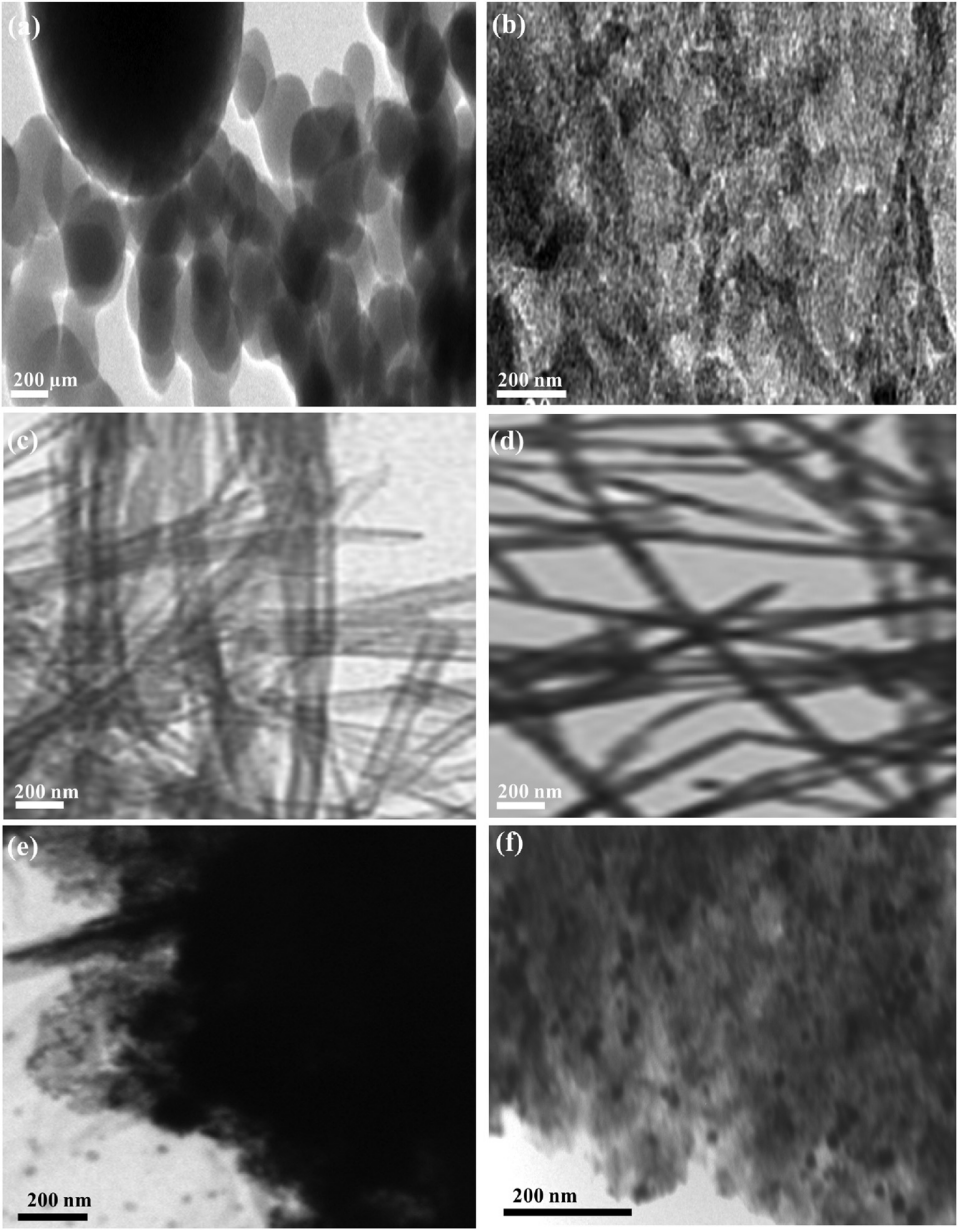
(a) TEM images of Pd/Cu/gC_3_N_4_NTs obtained by (a) quick addition of melamine, (b) quick addition of HNO_3_, (c) using 60 mL of HNO_3_ (0.03 M), (d) using ethanol instead of ethylene glycol. (e) Pd/Cu/gC_3_N_4_NTs formed using 60 mM of K_2_PdCl_4_ and CuCl_2_ and (f) using 40 mM of K_2_PdCl_4_ and CuCl_2._

Fig. 3a shows the TEM image of Pd/Cu/C_3_N_4_NTs nanoflakes prepared by the quick mixing of melamine (1 g) in an aqueous solution of ethylene glycol solution (30 mL) involving K_2_PdCl_4_ (20 mM) and CuCl_2_ (20 mM) followed by the slow addition of HNO_3_ (60 mL of 0.1 M) under stirring. The obtained precipitate was washed with ethanol and dried at 80 °C for 12 h before annealing at 550 °C (5 °C/min) for 2 h under nitrogen. The TEM image reveals the formation of aggregated flakes-like Pd/Cu/C_3_N_4_NTs nanostructures obtained in a high yield with an average dimension of ∼ 250 nm.

Under the same conditions and parameters of nanoflakes, the quick addition of nitric acid produced sheet-like nanostructures. This arose from the quick deprotonation and polymerization process via rapid addition of nitric acid (Fig. 3b). Reducing the concertation of nitric acid to 0.03 M with fixing all other conditions and parameters formed aggregated and non-uniform Pd/Cu/C_3_N_4_ nanotubes (Fig. 3c). Using isopropanol solution instated of ethylene glycol led to the production of Pd/Cu/C_3_N_4_ nanofibers in line with our previous reports (Fig. 3d) [2]. The as-formed nanofibers were highly uniform with average dimensions of 1.5 ± 0.2 μm in length and 80 ± 3 nm in width.

Fig. 3e shows the formation of gC_3_N_4_ nanosheets decorated with aggregated Pd/Cu nanoparticles formed through increasing the concertation of Pd/Cu to 60 mM instead of 20 mM with fixing all other conditions. Similarly, decreasing the concertation of Pd/Cu to 40 mM drove the formation of nanosheets decorated with uniform Pd/Cu nanoparticles (Fig. 3f). These results warranted that the formation of Pd/Cu/C_3_N_4_NTs is highly sensitive to the concentration of reactants and their mixing conditions. In particular, the addition of melamine and nitric acid should be sluggish to provide enough time for a consistent polymerization into uniform nanotubes. Nitric acid facilitates the deprotonation of active –NH_2_ groups of melamine and allowing the conversion of melamine into melem and then to polymeric gC_3_N_4_ composed of triazine-based units after carbonization at an elevated temperature [1], [2], [3], [4], [5]. Meanwhile, the concertation of Pd/Cu precursors should be lower to be anchored on the N-atoms of melamine and then facilitating the atomic doping of Pd/Cu instead of formation of nanoparticles [1], [2], [3], [4], [5]. On the other hand, glycol-mediated solution acting as a structure-directing agent for driving the formation of nanotube shape.

### 2.4 CO oxidation stability tests

**Fig. 4:**
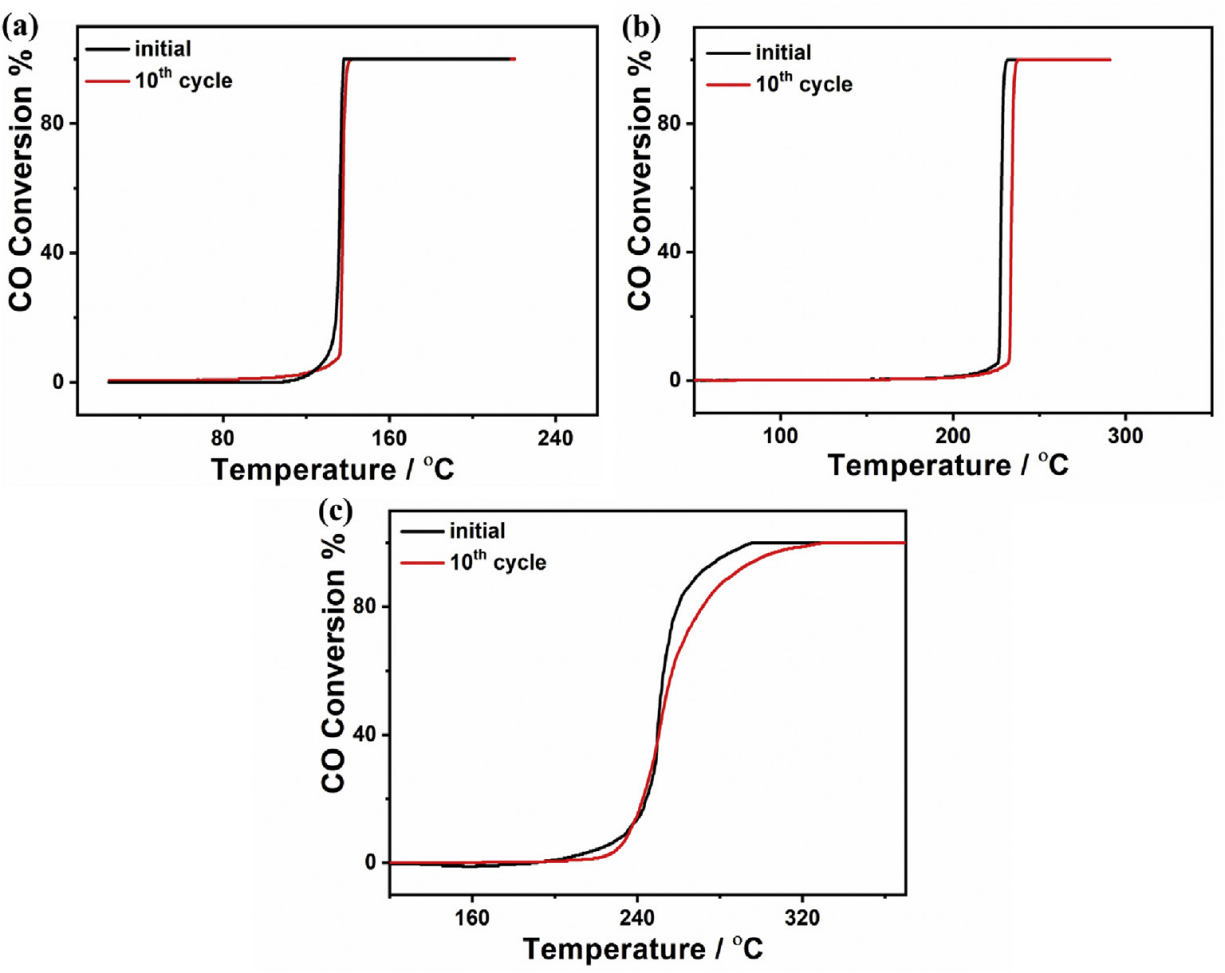
The CO oxidation light-off stability tests measured on (a) Pd/Cu/gC_3_N_4_NTs, (b) Pd/gC_3_N_4_NTs, and (c) Cu/gC_3_N_4_NTs for ten cycles at their T_100_.

The CO oxidation durability is an important factor in large-scale environmental and industrial applications [1], [2], [3], [4]. Fig. 4 shows the accelerated durability tests of Pd/Cu/gC_3_N_4_NTs, Pd/gC_3_N_4_NTs, and Cu/gC_3_N_4_NTs measured for ten cycles at their complete CO conversion temperature (T_100_). The results show that Pd/Cu/gC_3_N_4_NTs is more durable than both Pd/gC_3_N_4_NTs and Cu/gC_3_N_4_NTs. Particularly, the CO oxidation kinetics and T_100_ of Pd/Cu/gC_3_N_4_NTs were almost maintained without any significant changes (Fig. 4a). Meanwhile, the T_100_ of Pd/gC_3_N_4_NTs, and Cu/gC_3_N_4_NTs increased only by around 11 °C (Fig. 4b) and 25 °C (Fig. 4c), respectively. However, the CO oxidation kinetics did not decrease substantially on both Pd/gC_3_N_4_NTs and Cu/gC_3_N_4_NTs, as shown in their light-off curves (Fig. 4b and c).

**Fig. 5:**
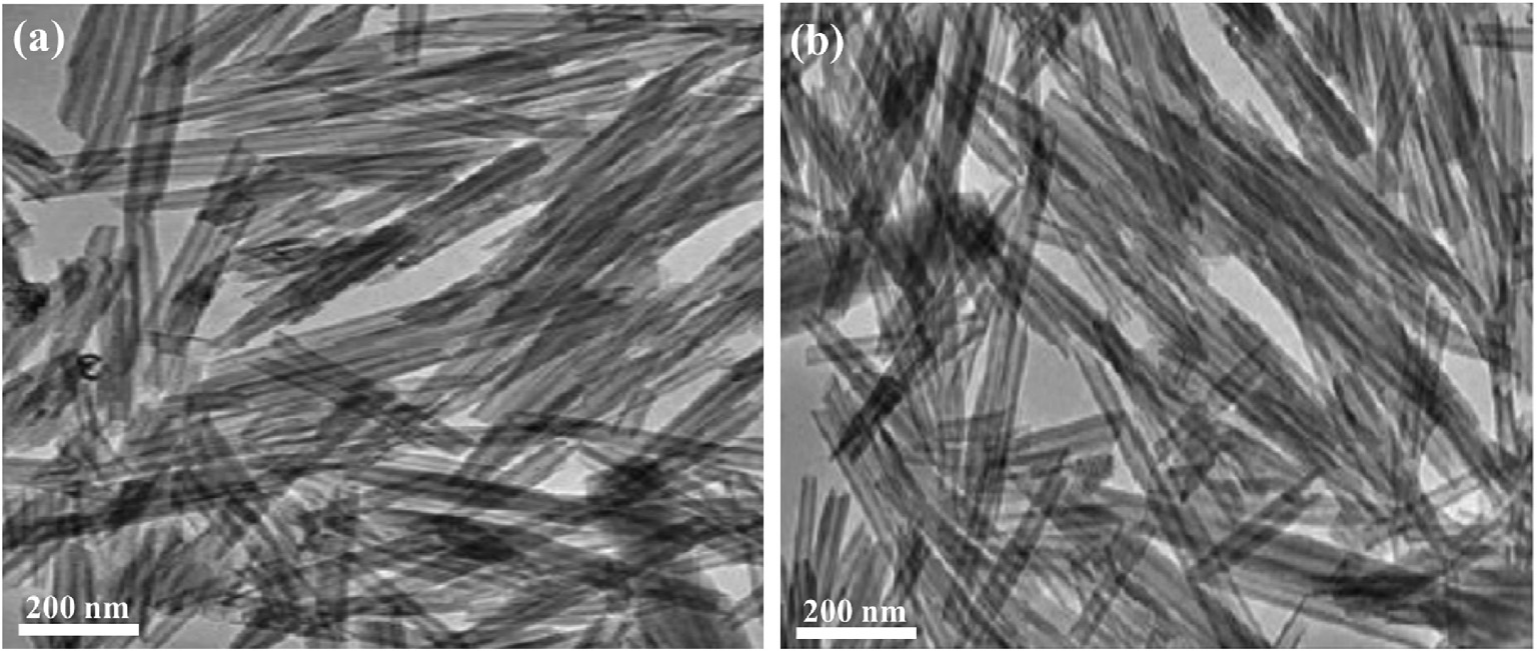
The TEM image of Pd/Cu/gC_3_N_4_NTs before (a) and after (b) the stability tests.

The sample was dispersed in ethanol and sonicated for 3 min and then mounted on a carbon-coated TEM grid. Fig. 5 reveals the TEM images of Pd/Cu/gC_3_N_4_NTs before (Fig. 5a) and after the CO oxidation stability tets (Fig. 5b). Comparing the TEM image of Pd/Cu/gC_3_N_4_NTs before and after the CO oxidation durability testes, we found that the structural stability of nanotube shape is fully maintained without any changes. Therefore, the nanotube morphology did not change after ten durability cycles.

**Fig. 6:**
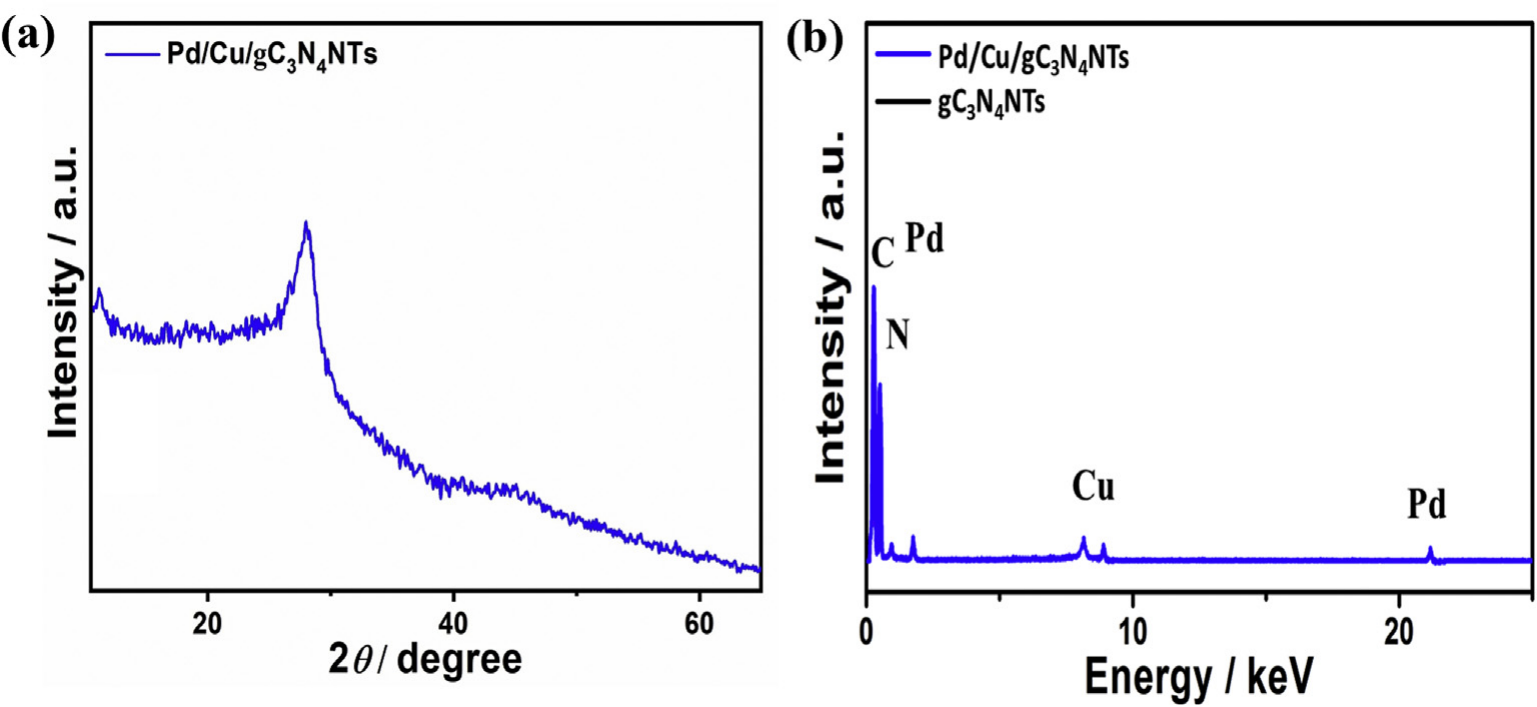
(a) XRD analysis and (b) EDX analysis of Pd/Cu/gC_3_N_4_NTs after the CO durability tests.

Fig. 6a shows the XRD analysis of Pd/Cu/gC_3_N_4_NTs after the CO durability tests, which displayed the one diffraction peak at 27.01° assigned to {002} facet and one peak at 13.15° attributes to{100} facet of gC_3_N_4_ nanostructure similar to those obtained for Pd/Cu/gC_3_N_4_NTs before the CO durability tests. Thus, the XRD result indicates that Pd/Cu/gC_3_N_4_NTs reserved its crystallinity after the CO oxidation durability tests. The EDX analyses after CO stability testes is carried out to confirm the compositional durability of Pd/Cu/gC_3_N_4_NTs (Fig. 6b). The results showed the presence of C, N, Pd, and Cu with atomic contents of 58, 40.9, 0.5, and 0.6, respectively (Fig. 6b). Thus, the EDX result implies that Pd/Cu/gC_3_N_4_NTs kept its composition without any deterioration, owing to the homogenous distribution of Pd and Cu inside the carbon matrix.

Table 1 shows the comparison between the catalytic CO oxidation activity of our newly designed Pd/Cu/gC_3_N_4_NTs and the previously reported catalysts such as Pd-based, Au-based Cu-based, Pt-based, and Mn-based. The complete conversion temperature of our obtained Pd/Cu/gC_3_N_4_NTs was significantly lower than that of all the catalysts reported in the literature as shwon in Table 1 in addition to the low cost of our catalyst that was made of nearly 99% gC_3_N_4_NTs and 1% Pd/Cu.

**Table 1:** Comparison between the CO oxidation activity of our newly designed Pd/Cu/gC_3_N_4_NTs and various catalysts reported elsewhere.

Catalyst	Complete CO conversion, T_100_	Reference
Pd/Cu/gC_3_N_4_NTs	154 °C	Our work
Au_0.75_Cu_0.25_/SiO_2_	300 °C	[6]*Catal. Today,* 2017, 282 105–110.
Pd/La-doped γ-alumina	175 °C	[7]*Nat. Commun.*, 2014, *5*, 4885.
Pd-impeded 3D porous graphene	190 °C	[8]*ACS Nano* 2015, 9, 7343-7351
Pt/CNx/SBA-15	250 °C	[9]*Chem. A Eur. J.*, 2014, 20, 2872–2878.
Nanoarray-based CuMn_2_O_4/_Washed-coated CuMn_2_O_4_	320 °C/350 °C	[10]*J. Mater. Chem. A*, 2018, 6, 19047-19057
Cu_1_/Mn_1_	180 °C	[11]*Catal. Lett.,* 2016, 146, 2364-2375
MnO_x_	310 °C	[12]*Catal. Sci. Technol.*, 2016, 6, 8222-8233

The TEM, XRD, and EDX results confirmed the structural and compositional stability of the as-synthesized Pd/Cu/gC_3_N_4_NTs after the CO oxidation stability tests. This probably originates from coupling between the unique physicochemical properties of 1D gC_3_N_4_ nanotubes (e.g., stability, massive accessible active sites, thermal stability nearly up to 600 °C, and chemical stability in various solvents) and the inherent catalytic merits of Pd/Cu (eg., electronic effect, synergetic effect, strong adsorption/activation/dissociation for CO/O_2_, and high tolerance for CO_2_ product) [1], [2], [3], [4], [5], [13], [14], [15], [16]. Chemically speaking, the atomic doping of gC_3_N_4_NTs with Pd and Cu stabilizes them against aggregation as well as protecting their active catalytic sites from the blocking by the reaction intermediates or products.

**Scheme 2:**
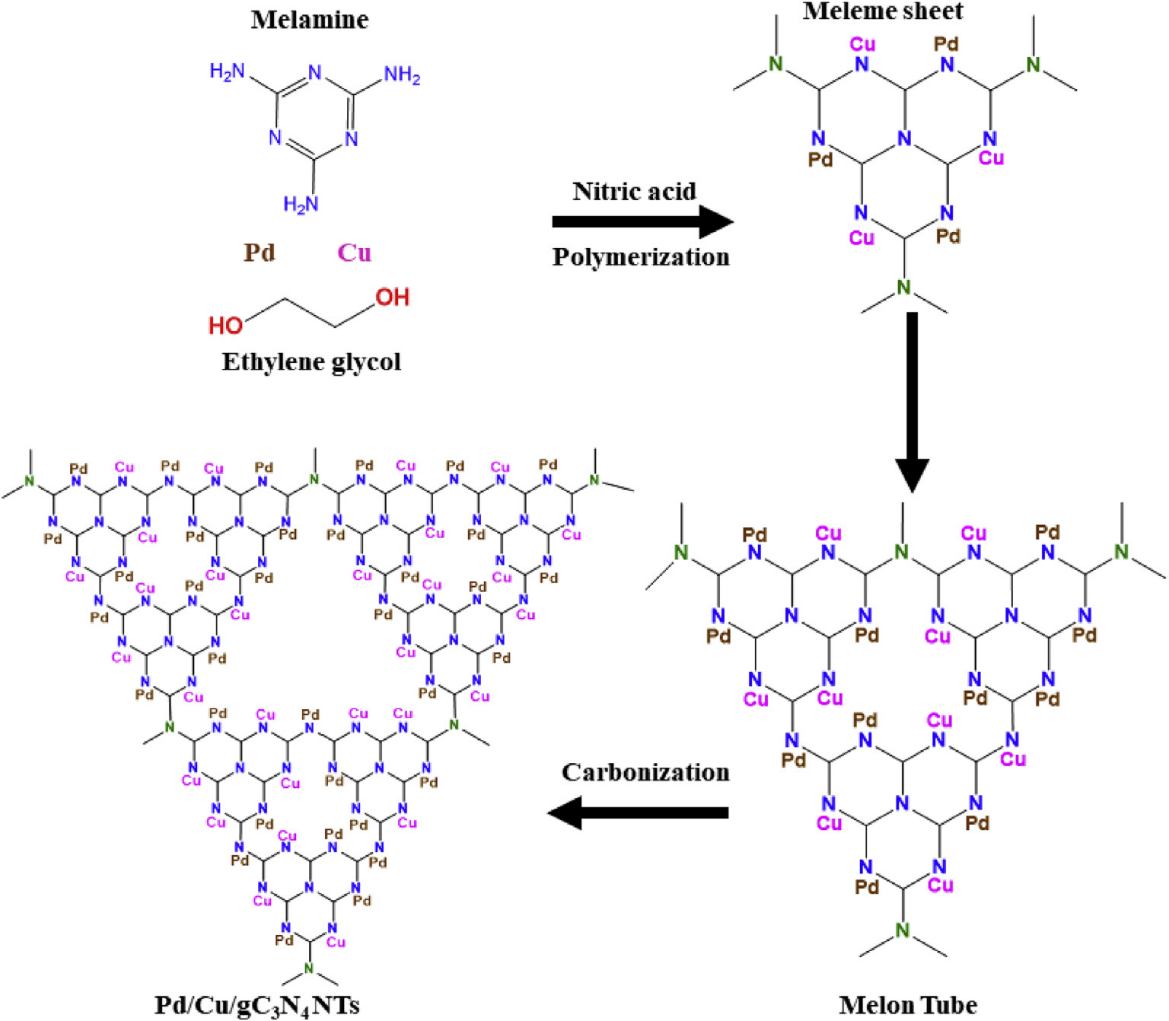
The formation mechanism of Pd/Cu/gC_3_N_4_NTs and the distribution of Pd and Cu inside gC_3_N_4_NTs.

Scheme 2 shows the formation process and mechanism of typically prepared Pd/Cu/gC_3_N_4_. The strong binding affinity between N-atoms of melamine and Pd/Cu facilitate adsorption and anchoring of both Pd and Cu on N-atoms during the polymerization step that led to the homogenous atomic distribution of Pd, and Cu on the N-atoms of gC_3_N_4_.

**Fig. 7:**
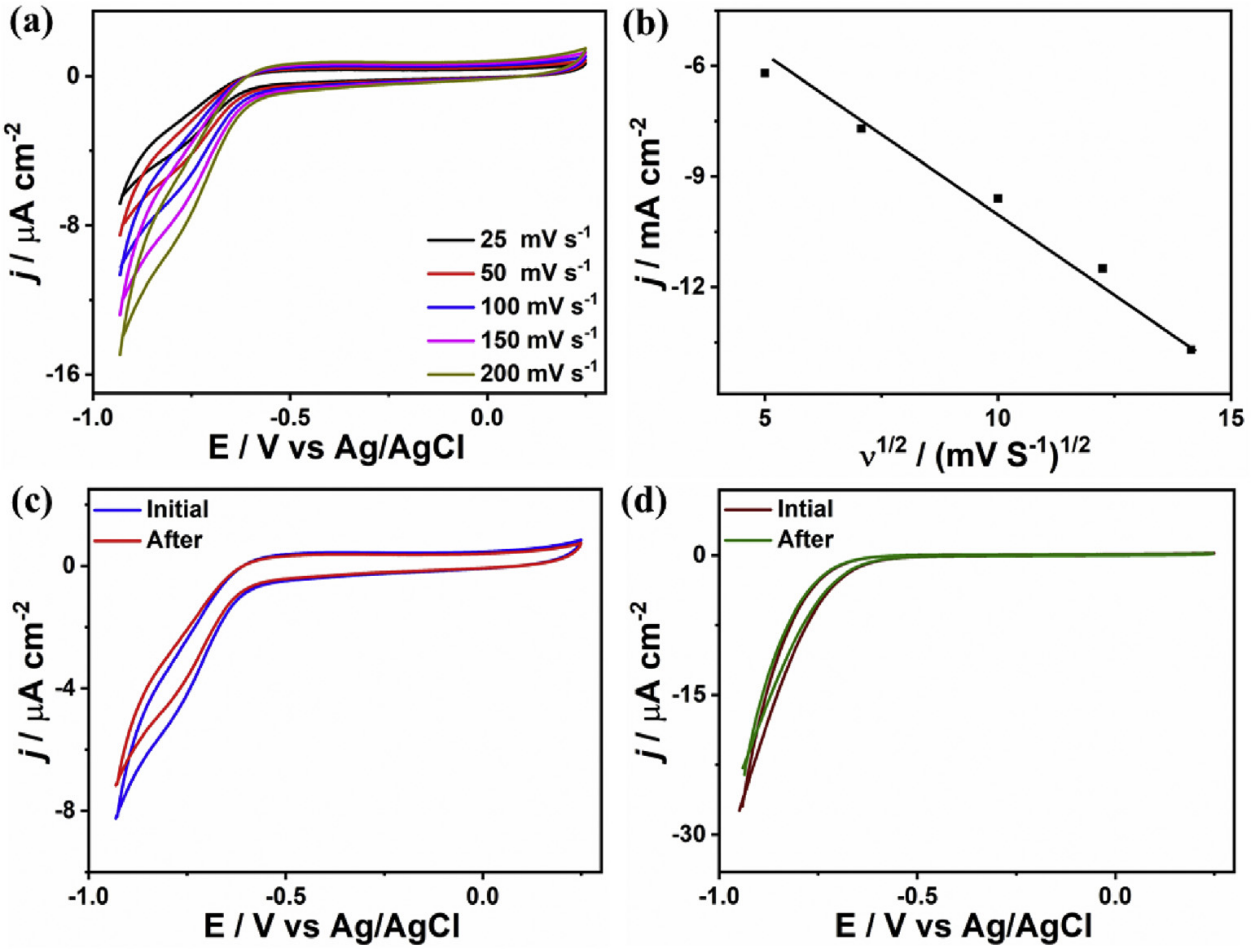
(a) The CVs measured on the as-made catalysts in CO_2_-saturated 0.5 NaHCO_3_ at 50 mV s^−1^ under different scan rates and (b) Randles-Sevcik equation. (c) The electrochemical CVs durability on Pd/Cu/gC_3_N_4_NTs measured under dark. (d) The photoelectrochemical CVs stability tested under continuous light irradiation (100 W). We performed all the measurements at the room temperature.

Fig. 7a shows the CVs for CO_2_ reduction measured under various sweeping rates ranged from 25 to 200 mV s^−1^, which showed the steady enhancement in the current density with increasing the scan rate. The relationship between the obtained current densities and the square root of scan rates is linear (Fig. 7b).

The electrocatalytic and photo-electrochemical CO_2_ reduction durability tests were carried out on Pd/Cu/gC_3_N_4_NTs via measuring the chronoamperometric test (*I*-*T*) for 30 min in CO_2_-saturated an aqueous solution of 0.5 NaHCO_3_ at 50 mV s^−1^. Then the CVs curve were measured again in CO_2_-saturated an aqueous solution of 0.5 NaHCO_3_ at 50 mV s^−1^. The CVs curves showed that Pd/Cu/gC_3_N_4_NTs kept its initial electrocatalytic CO_2_ reduction activity (Fig. 7c) without any significant deterioration in the current density, reduction kinetics, and reduction potential (Fig. 7d), [17].

**Fig. 8:**
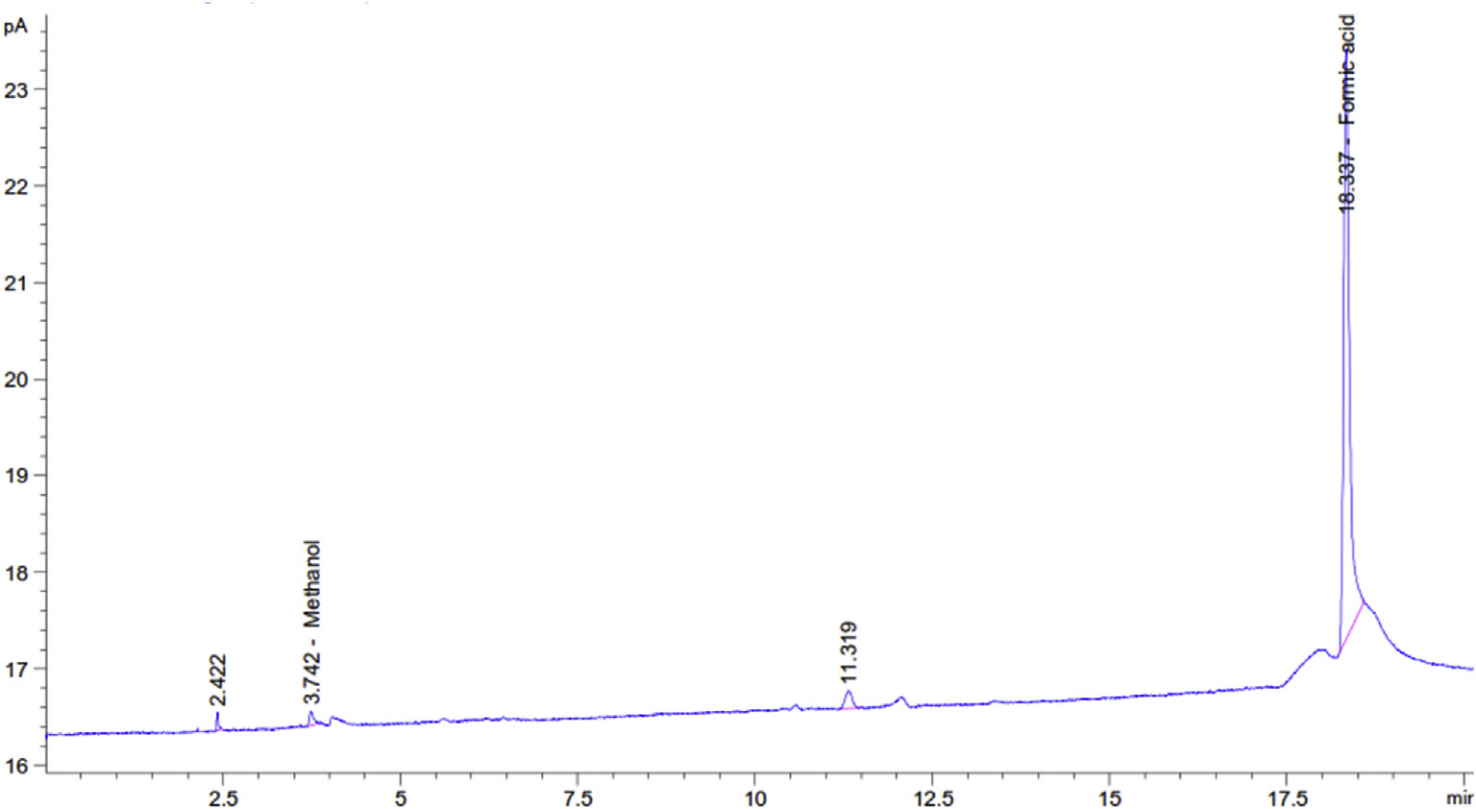
CO_2_ reduction products obtained from the Gas chromatography (Agilent Technologies 7890A) with using a column PerkinElmer Elite-624 at 35 °C.

Fig. 8 depicts the gas chromatography result that was obtained after calibration relative to pure formic acid and methanol under the same conditions. The results demonstrated the presence of formic acid as the main product as well as methanol as an inferior product (Fig. 8). Therefore, the gas chromatography indicates the ability of Pd/Cu/gC_3_N_4_NTs to reduce CO_2_ electrochemically to formic acid at room temperature.
